# Pan-genome Analyses of the Species *Salmonella enterica*, and Identification of Genomic Markers Predictive for Species, Subspecies, and Serovar

**DOI:** 10.3389/fmicb.2017.01345

**Published:** 2017-07-31

**Authors:** Chad R. Laing, Matthew D. Whiteside, Victor P. J. Gannon

**Affiliations:** National Microbiology Laboratory, Public Health Agency of Canada Lethbridge, AB, Canada

**Keywords:** genomics, pan-genome, *Salmonella*, predictive markers, food safety

## Abstract

Food safety is a global concern, with upward of 2.2 million deaths due to enteric disease every year. Current whole-genome sequencing platforms allow routine sequencing of enteric pathogens for surveillance, and during outbreaks; however, a remaining challenge is the identification of genomic markers that are predictive of strain groups that pose the most significant health threats to humans, or that can persist in specific environments. We have previously developed the software program Panseq, which identifies the pan-genome among a group of sequences, and the SuperPhy platform, which utilizes this pan-genome information to identify biomarkers that are predictive of groups of bacterial strains. In this study, we examined the pan-genome of 4893 genomes of *Salmonella enterica*, an enteric pathogen responsible for the loss of more disability adjusted life years than any other enteric pathogen. We identified a pan-genome of 25.3 Mbp, a strict core of 1.5 Mbp present in all genomes, and a conserved core of 3.2 Mbp found in at least 96% of these genomes. We also identified 404 genomic regions of 1000 bp that were specific to the species *S. enterica*. These species-specific regions were found to encode mostly hypothetical proteins, effectors, and other proteins related to virulence. For each of the six *S. enterica* subspecies, markers unique to each were identified. No serovar had pan-genome regions that were present in all of its genomes and absent in all other serovars; however, each serovar did have genomic regions that were universally present among all constituent members, and statistically predictive of the serovar. The phylogeny based on SNPs within the conserved core genome was found to be highly concordant to that produced by a phylogeny using the presence/absence of 1000 bp regions of the entire pan-genome. Future studies could use these predictive regions as components of a vaccine to prevent salmonellosis, as well as in simple and rapid diagnostic tests for both *in silico* and wet-lab applications, with uses ranging from food safety to public health. Lastly, the tools and methods described in this study could be applied as a pan-genomics framework to other population genomic studies seeking to identify markers for other bacterial species and their sub-groups.

## Introduction

The global burden of bacterial enteric disease, much of it foodborne, results in an estimated 2.2 million deaths per year, and an annual loss of 112,000 disability adjusted life years in the United States alone (Bergholz et al., 2014; Scallan et al., 2015). Nationwide molecular diagnostic networks, such as PulseNet in North America, were designed to enable the rapid identification of outbreaks by genetic fingerprinting the etiological agents of disease, and keeping nationwide databases of genetic fingerprints of specific pathogens associated with human disease. Since its inception, PulseNet has relied on pulsed-field gel electrophoresis (PFGE) for fingerprinting of bacterial pathogens to identify the specific sources of outbreaks and prevent further infections. Using this approach, it has been estimated that PulseNet prevents 277,000 illnesses from bacterial pathogens annually in the United States, reducing the costs associated with medical care and loss of productivity due to worker illness (Scharff et al., 2016).

Despite the usefulness of PulseNet, the PFGE technique itself is often unable to distinguish between related and unrelated strains, due to its reliance on rare-cutting restriction enzyme sites within the genome (Allard et al., 2012). Additionally, the interpretation of the banding patterns among labs requires extensive training and standardization to enable meaningful comparisons. Lastly, the banding patterns provide no information on the actual content of the genomes they represent, so important information regarding human virulence, such as the presence or absence of known toxins, is not available.

Lastly, while the presence of known virulence factors has been correlated with severe human disease in a number of bacterial species, it has also been shown that some lineages or clades within these same species, while possessing specific virulence factors, are rarely associated with human disease (Lupolova et al., 2016; Waryah et al., 2016). Thus, multiple virulence factors, and regulatory genes that influence the expression of key virulence factors, or otherwise modulate the virulence of these strains, need to be taken into consideration when attempting to predict the strains of a bacterial species that are potential human health threats (Opijnen et al., 2012).

Recently, whole-genome sequencing (WGS) has displaced PFGE as the *de facto* standard for the complete characterization of bacterial pathogens, in both ongoing surveillance and outbreak investigations (Deng et al., 2016; Franz et al., 2016). WGS allows clear definition between outbreak-related strains and those from unrelated sources, and it has the ability to identify routes of transmission, and attribute bacterial contaminants to specific sources (den Bakker et al., 2014). It is currently being utilized in reference laboratories worldwide. Examples of its application include the sequencing of all *Listeria monocytogenes* isolated in the United States, all *Salmonella* isolated by the Food and Drug Administration in the USA, and by Public Health England as part of routine surveillance (Ashton et al., 2016), and a large-scale survey of *Staphylococcus aureus* in continental Europe. In the latter study, the applicability of WGS for the identification of the emergence and spread of clinically relevant *Staphylococcus aureus* was demonstrated (Aanensen et al., 2016).

It has also recently been shown that antimicrobial resistance (Tyson et al., 2015; McDermott et al., 2016; Zhao et al., 2016), serovar (Levine et al., 2016; Yoshida et al., 2016b), and the results of other traditional sub-typing schemes such as multi-locus sequence typing (Sheppard et al., 2012) can be accurately predicted *in silico* through the analysis of bacterial genome sequences. However, identifying bacterial isolates that are most likely to cause disease in humans, based on the genome sequence alone, is a more complex task. In addition, markers that can identify bacteria likely to exhibit particular phenotypes, such as the ability to survive in a particular niche, or the ability to tolerate harsh environments such as those found in food processing plants are also required.

We have previously developed the software platform Panseq, for the analyses of thousands of genomes in a pan-genome context, where both the presence/absence of the accessory genome and SNPs within the shared core-genome are computed (Laing et al., 2010). Additionally, we recently released a platform for the predictive genomics of *Escherichia coli*, called SuperPhy, in which markers statistically biased within groups of bacteria, based on any metadata category, can be identified (Whiteside et al., 2016).

In this study we use our previously created software to examine the pan-genome of *Salmonella enterica*, a pathogen that causes an estimated 93.8 million cases of enteric illness worldwide each year (Majowicz et al., 2010; Gal-Mor et al., 2014). The species *S. enterica* is divided into six subspecies: *enterica*, *salamae*, *arizonae*, *diarizonae*, *houtenae*, and *indica*. Over 99% of human disease caused by *S. enterica* is done so by subspecies *enterica*, with the World Health Organization estimating that *S. enterica* infections from contaminated food alone constitute a loss of 6.43 million disability adjusted life years worldwide, more than any other enteric pathogen (Kirk et al., 2015). Within this bacterial subspecies, are human-adapted strains responsible for typhoid fever, as well as a large number of animal-derived non-typhoidal strains responsible for foodborne illness. In this study, we have identified species- and subspecies-specific markers, as well as markers predictive of serovar for subspecies enterica. While this study focused on *S. enterica*, the tools and approach are broadly applicable to any species or collection of genomes.

## Materials and Methods

All commands and parameters used to analyze the data and generate the Figures are available as Supplementary File 1. The scripts used for analyses are available at https://github.com/superphy/gamechanger. The following is a summary of the methods used.

### Data Collection

All *S. enterica* genomes were downloaded from GenBank in nucleotide fasta format. A full listing of the initial 4939 genomes, including GenBank identifier, subspecies, serovar, the number of species-specific core regions present, the number of contigs, and whether the genome passed the quality filtering steps are listed in Supplementary File 2.

### Serovar Identification

Most of the *S. enterica* genomes in GenBank had serovar provided as part of their metadata; however, 321 were missing this designation. The SISTR web-server, as well as the SISTR commandline app were used to predict the serovar for these strains (Yoshida et al., 2016b).

### Pan-genome Analyses

Panseq (commit:1d0ab9d37e8e358d266e1d0aa80e9b27f28a1def) was used to identify the pan-genome of the 4939 strains in this study (Laing et al., 2010). Genomes were initially fragmented into 1000 bp segments, and subsequently clustered using cd-hit v.4.6 to remove potential duplicates/paralogs from the analyses using a 90% sequence identity threshold (Fu et al., 2012). Initially Panseq was used to determine the distribution of the pan-genome among the genomes at a 90% sequence identity threshold, from which a “conserved core” was identified. Within the conserved core, Panseq was then used to identify single-nucleotide polymorphisms.

### Identification of *S. enterica* Species-Specific Regions

To identify regions that were likely to represent the species as a whole, we initially examined the 211 closed *S. enterica* genomes in GenBank (Supplementary File 2), and identified 3832 regions of 1000 bp that were found in 90% (190) of the 211 closed genomes using Panseq, at a 90% sequence identity threshold. These regions were then screened against the online GenBank nr database using megablast as a first-pass filter with default parameters, searching across bacteria (taxid:2), and excluding all *Salmonella* (taxid:590) hits that had greater than 80% identity across 80% of the query length from the results. The remaining 1482 genomic regions were subsequently screened against the online GenBank nr database of all bacteria (taxid:2), using the blastn algorithm, to identify matches that were missed using the less-specific megablast algorithm, with word size 11, an e-value cutoff of 0.001, and excluding all *Salmonella* (taxid:590). These results were filtered in the same manner, leaving 405 potentially species-specific regions. Lastly, these regions were compared against *Salmonella bongori* genomes in GenBank; one *S. bongori* hit was identified, which left 404 genomic regions present in *S. enterica* but no other bacterial genomic sequences within the GenBank nr database.

The putative function of these regions was determined by screening them across the GenBank nr database using blastx with “max hits:10,” “taxid limit:1236 (gammaproteobacteria),” and an “e-value threshold: 0.001.” The best matching hit above a 90% sequence identity threshold was used for the putative functional assignment.

### Identification of Subspecies- and Serovar-Specific Regions

The Fisher’s Exact test, using the Bonferroni correction for multiple testing was applied as in the SuperPhy platform (Whiteside et al., 2016), implemented here as the standalone program feht^1^. The input for the program was Supplementary File 2, which contained metadata for all the strains, as well as the binary_table.txt output file from the Panseq analyses, which denotes the presence/absence of each 1000 bp pan-genome region among all the strains.

### *S. enterica* Phylogenetic Analyses

The phylogeny based on SNPs within the core genome was generated using RAxML v8.2.9, with the snp.phylip output file from Panseq (Stamatakis, 2014). The phylogeny based on the presence/absence of the pan-genome was also generated using RAxML v8.2.9, with the binary.phylip output file from Panseq.

### Generation of Figures and Tables

The R-statistical language v3.3.2 was used to generate the summary Figures and Tables (R Core Team, 2016). The R-scripts and all others used for the analyses can be found at https://github.com/superphy/gamechanger/tree/master/src. The ggtree package for R was used in the generation of the phylogenetic tree images (Yu et al., 2016).

## Results

### *S. enterica* Pan-genome

**FIGURE 1:**
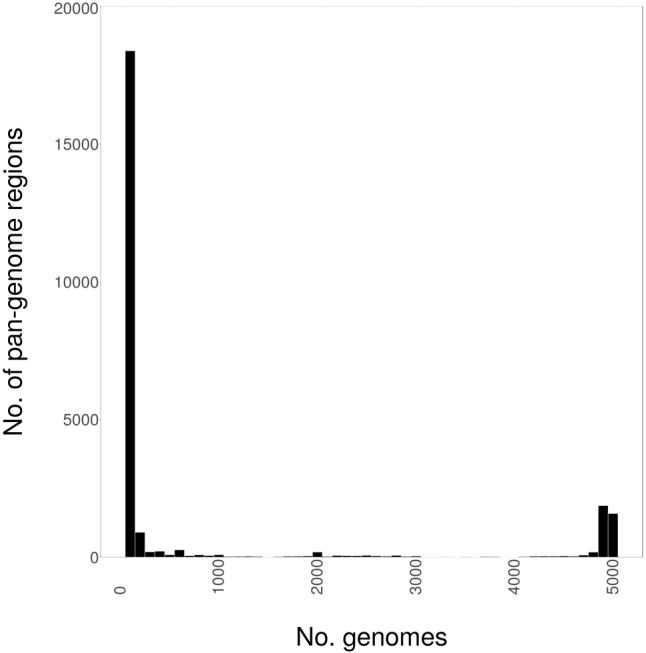
The distribution of the *Salmonella enterica* pan-genome, as 1000 bp fragments, among 4939 whole-genome sequences (WGSs).

We initially determined the size and distribution of the *S. enterica* pan-genome as genome fragments of 1000 bp in size, across the 4939 genome sequences of this study, which are summarized by subspecies in **Table 1**, and within subspecies *enterica* by serovar in **Table 2**. As can be seen in **Figure 1**, the pan-genome comprised of 4939 *S. enterica* genomes was found to be 25.3 Mbp in size, with 70% of the pan-genome present in fewer than 100 strains. Conversely, the core genome was found to be 1.5 Mbp in size, with all but 200 genomes (96%) containing 3.2 Mbp of shared genomic core. Only 17% of the pan-genome was found in greater than 100 genomes, but fewer than 4739 genomes.

**Table 1:** The frequency of the subspecies observed within the study set of 4936 *Salmonella enterica* genomes, prior to any quality filtering.

Subspecies	No.
*enterica*	4913
*arizonae*	7
*diarizonae*	7
*houtenae*	4
*salamae*	4
*indica*	1


**Table 2:** The serovars with more than 20 representatives in the current study set of 4936 *Salmonella enterica* genomes, and their frequency, prior to any quality filtering.

Serovar	No.
Typhi	1977
Typhimurium	758
Enteritidis	413
Heidelberg	201
Paratyphi	158
Kentucky	155
Agona	136
Weltevreden	120
Bareilly	106
Newport	82
Tennessee	77
Montevideo	69
Saintpaul	48
Infantis	39
Senftenberg	35
Bovismorbificans	34
Hadar	33
Muenchen	30
Anatum	27
Schwarzengrund	27
Dublin	24
Cerro	21


*The list of all serovars and their frequency within the current study is available as Supplementary File 2.*

### *S. enterica* Species-Specific Regions

To identify regions of *S. enterica* that were likely to be shared among most genomes of the species, we examined all 211 closed genomes of *S. enterica* in GenBank, looking for genomic regions that were present in at least 190 (90%) of these genomes. We identified 3832 regions of 1000 bp that were present in at least 90% of the closed genomes. These regions were subsequently screened against the GenBank nr database, and any present in non-*Salmonella* genomes were removed, leaving 404 putative *S. enterica* species-specific regions (Supplementary File 3).

**FIGURE 2:**
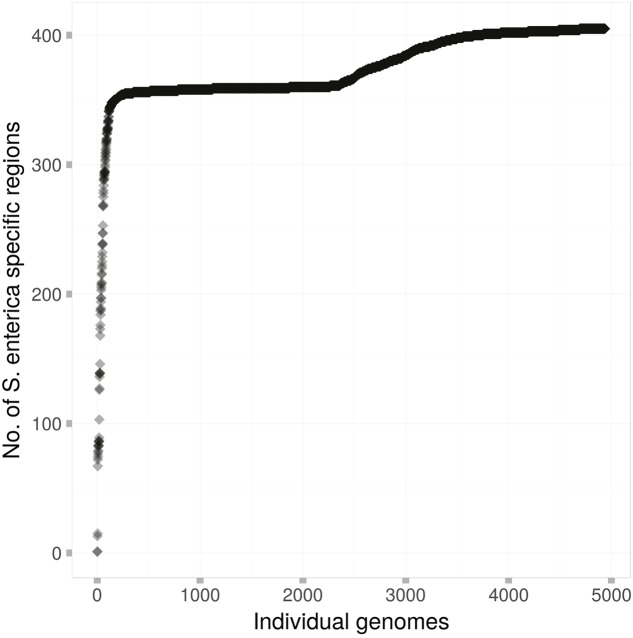
The carriage of the 404 *S. enterica* species-specific regions among each of the 4939 genomes of this study. Each dot represents a single *S. enterica* genome, which are arranged in order from those that contain the fewest species-specific regions to those that contain the most.

**Figure 2** shows the carriage of these 404 regions among the 4939 genomes of this study. All but 105 genomes contained at least 330 of these putative *S. enterica* specific regions. A stark difference in carriage of these species-specific markers was observed, with 4742 genomes containing at least 350 species-specific markers, while only 2674 genomes contained 360 or more species-specific markers.

### Quality Filtering for Subsequent Analyses

**FIGURE 3:**
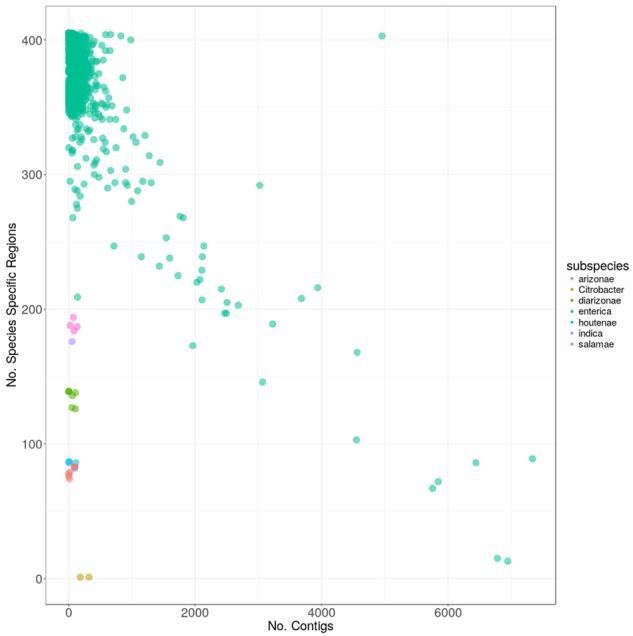
The carriage of the 404 *S. enterica* species-specific regions, versus the number of contigs for each of the 4936 genomes. Colors indicate the subspecies within *S. enterica* as follows: red: arizonae, lime: diarizonae, teal: enterica, blue: houtenae, lavender: indica, magenta: salamae and yellow: sample with *Citrobacter* contamination.

To ensure the quality of the genomes in use for subsequent analyses, we plotted carriage of the 404 species-specific regions versus the number of contigs that each sequenced genome was comprised of (**Figure 3**). As can be seen, the two genomes marked in yellow contained only one, and the same, species-specific region each, despite being comprised of relatively few contigs. Subsequent searches against the GenBank nr database identified these two genomes as *Citrobacter* spp. contamination, mislabeled as *S. enterica* (GCA_001570325 and GCA_001570345). The “*Salmonella enterica* species-specific region” found in both of the contaminant *Citrobacter* genomes, did not match any other *Citrobacter* spp. in GenBank above the thresholds used for determining presence/absence in this study. However, due to the presence of this region in what have been identified as *Citrobacter* genomes, the region was removed from subsequent analyses.

The majority of genomes (4913) were from subspecies *enterica*, with genomes from the five other *S. enterica* subspecies present in drastically fewer numbers (**Table 1**). All closed genomes from subspecies *enterica* contained greater than 250 species-specific regions, which was more than the genomes from any other subspecies, with the exception of *enterica* genomes that were of poor quality and comprised of many 1000s of contigs (**Figure 3**). Genomes from subspecies *houtenae* and *arizonae* contained fewer than 100 species-specific regions, while genomes from *diarizonae*, *indica*, and *salamae* contained between 100 and 200 species-specific regions. All regions were screened against *S. bongori* to ensure specificity to *S. enterica*; one region was found to also be present in genomes from *S. bongori* and was removed from further analyses.

Within subspecies *enterica*, a negative linear relationship was observed among the number of species-specific regions contained within a genome, and the number of contigs the genome was comprised of, with the worst-case genome (GCA_000495155) being comprised of 6945 contigs, but containing only 13 species-specific regions. Other genomes such as *S. enterica* Bovismorbificans strain GCA_001114865 contained both few contigs (140) as well as fewer species-specific regions (209) than other *enterica* genomes. Additional searches discovered sequencing gaps within the genome totaling over 464 Kbp. A final outlier genome harbored nearly 5000 contigs, but also contained 403 of the species-specific regions. It was determined by searching the GenBank database, that this sequence (GCA_000765055) was actually a combination of multiple genomes in a single file.

Given the above information, all genomes from the five subspecies other than *enterica* were included in subsequent analyses, while the thresholds for inclusion of *enterica* genomes were set at a maximum of 1000 contigs, and a minimum of 250 species-specific regions. Following this quality filtering, 43 genomes were removed, leaving 4870 *S. enterica* subspecies *enterica* genomes for the following analyses.

### Phylogeny of *S. enterica* Using the Conserved Core Genome

**FIGURE 4:**
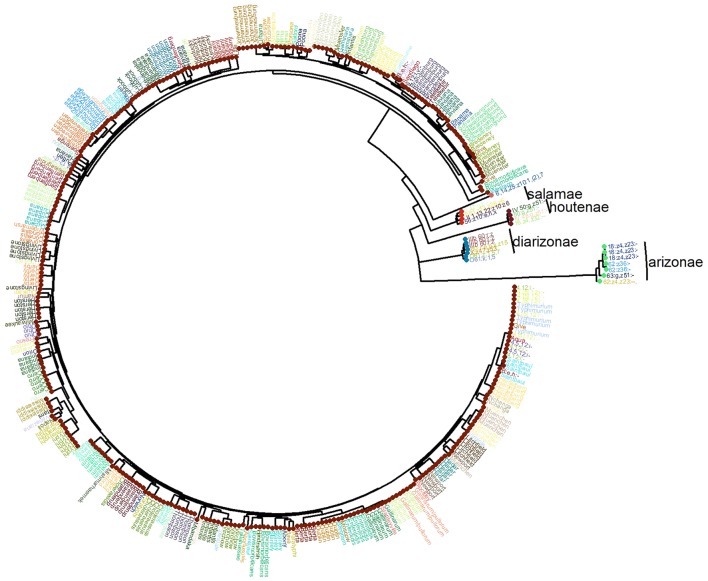
The phylogeny of the 4893 *S. enterica* genomes post quality-filtering, and limiting the number of genomes from each serovar to five. The name of each serovar is presented as text, and the six subspecies are shown as colored circles as follows: teal: arizonae, blue: diarizonae, dark orange: enterica, peach: houtenae, dark green: indica, light orange: salamae.

Based on the distribution of the pan-genome presented in **Figure 1**, the “conserved core” of *S. enterica* was set at being present in more that 4500 genomes, to fully capture the conserved genomic regions within the species. A phylogeny based on the SNPs among these shared regions was created, and is shown along with the distribution of the *S. enterica* species-specific regions in **Figure 4**. As can be seen, the majority of the genomes are subspecies *enterica*, and the other five subspecies are relatively more distant in the order of *indica*, *salamae*, *houtenae*, *diarizonae*, and *arizonae*. However, the order of subspecies in declining number of species-specific regions is: *enterica*, *diarizonae*, *salamae*, *indica*, *houtenae*, and *arizonae*, which is shown in **Figure 3**.

**FIGURE 5:**
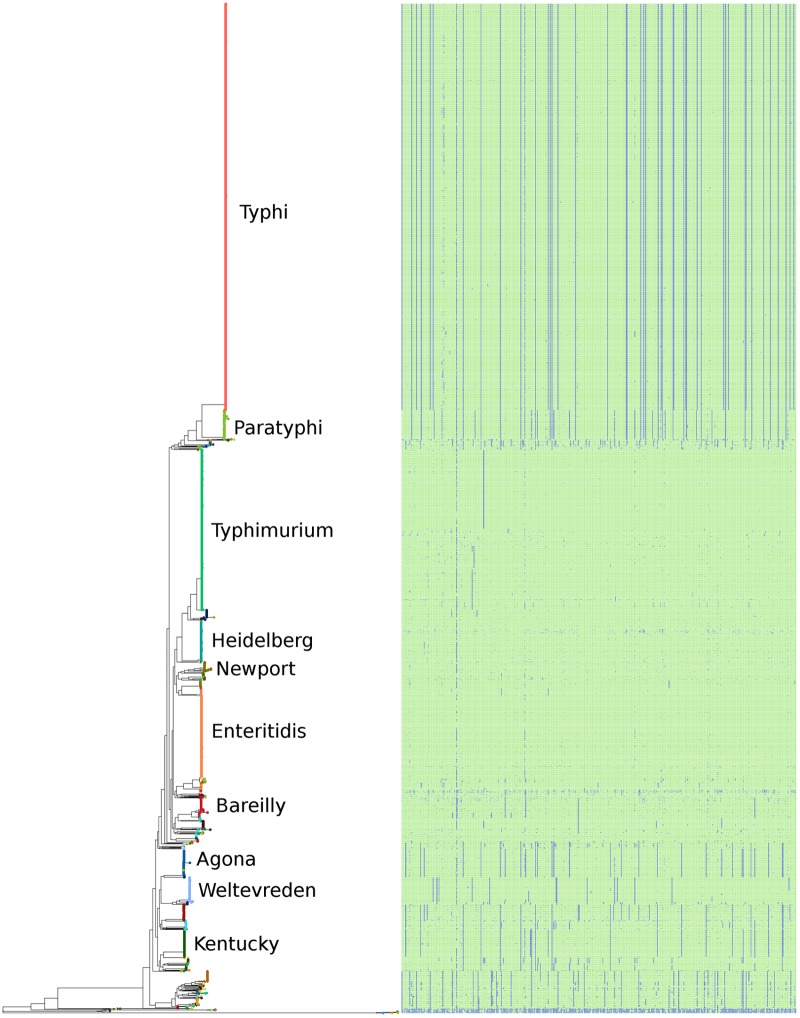
The phylogeny of the 4893 *S. enterica* genomes post quality-filtering based on SNPs found within the conserved core genome. The 10 most abundant serovars of subspecies enterica in the current study (Agona, Bareilly, Enteritidis, Heidelberg, Kentucky, Newport, Paratyphi, Typhi, Typhimurium, Weltevreden) are labeled on the tree. The matrix to the right of the phylogeny represents the 404 species-specific regions, with blue being the absence of a region, and green being the presence of a region, for each of the genomes of the study.

The serovar distribution within subspecies *enterica* was shown to be largely concordant with phylogeny, as demonstrated in **Figure 5**, where the 10 most abundant serovars in the current study are highlighted. However, not all serovars clustered as monophyletic groups, as can be seen with serovar Bareilly; nor were all clades found to be comprised of single serovars, demonstrated by the clade containing genomes of serovars Bareilly and Agona.

The large clades within the phylogenetic tree also demonstrate clade-specific patterns of presence/absence for the 404 species-specific markers. Among the most abundant serovars, Typhimurium, Heidelberg, Newport, and Enteritidis were found to contain the most species-specific markers, and grouped together near the center of the tree. Likewise, serovars Agona, Welevreden, and Kentucky contained fewer species-specific regions, and group together near the bottom of the tree, closer to the non-*enterica* sub-species genomes.

**Table 3** considers all serovars with at least 10 members in the dataset, and the average number of species-specific markers per serovar. As can be seen, the serovars with the largest average number of species-specific regions were: Enteritidis (401.7), Anatum (401.5), Muenchen (400.5), Hadar (400.3), and Typhimurium (400.1); conversely, the serovars with the fewest average number of species-specific regions were: Derby (360.7), Montevideo (360.1), Typhi (358.1), Bovismorbificans (355.3), and Cerro (342.0).

**Table 3:** The average number of species-specific genomic regions found among serovars of subspecies *enterica*, that contained at least 10 representative genomes, within the 4870 quality filtered subspecies *enterica* genomes of this study.

Serovar	Average no. species-specific regions
Enteritidis	401.7
Anatum	401.5
Muenchen	400.5
Hadar	400.3
Typhimurium	400.1
Newport	399.8
Thompson	399.7
Saintpaul	399.6
Heidelberg	397.4
Dublin	395.2
Infantis	394.9
Braenderup	392.8
Weltevreden	390.0
Bareilly	388.5
Kentucky	380.3
Plymouth/Zega	377.9
Senftenberg	376.5
Mbandaka	374.5
Lubbock	374.1
Reading	370.4
Agona	369.5
Tennessee	368.3
Schwarzengrund	362.3
Paratyphi	361.5
Derby	360.7
Montevideo	360.1
Typhi	358.1
Bovismorbificans	355.3
Cerro	342.0


### Phylogeny of *S. enterica* Using the Pan-genome

**FIGURE 6:**
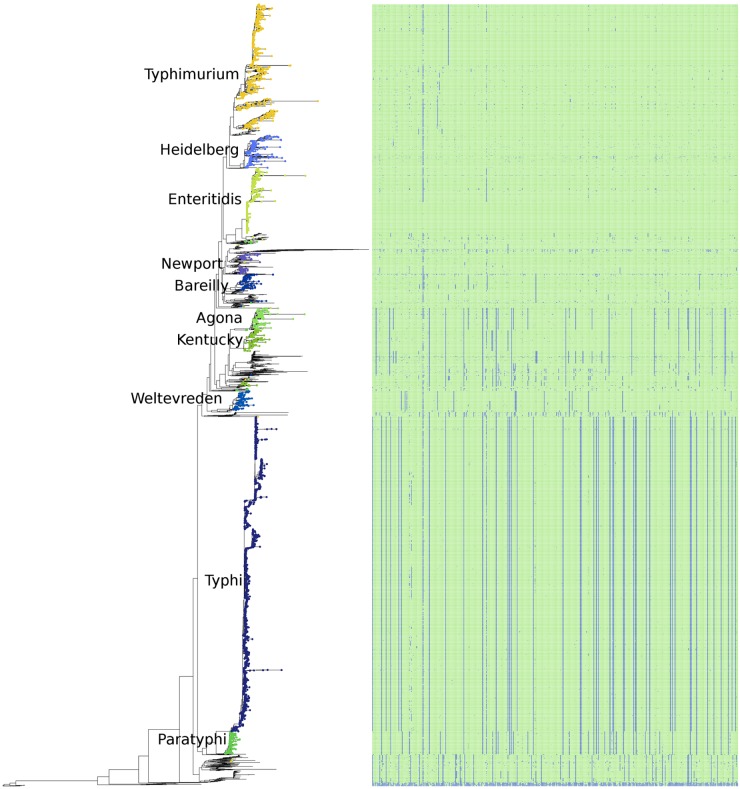
The phylogeny of the 4893 *S. enterica* genomes post quality-filtering based on the presence/absence of the entire pan-genome as 1000 bp fragments. The 10 most abundant serovars of subspecies enterica in the current study (Agona, Bareilly, Enteritidis, Heidelberg, Kentucky, Newport, Paratyphi, Typhi, Typhimurium, Weltevreden) are labeled on the tree. The matrix to the right of the phylogeny represents the 404 species-specific regions, with blue being the absence of a region, and green being the presence of a region, for each of the genomes of the study.

A phylogeny based on the presence/absence of the pan-genome among the 4893 *S. enterica* genomes was created, and is shown along with the distribution of the *S. enterica* species-specific regions in **Figure 6**. As can be seen this phylogeny based on the presence/absence of the entire 25.3 Mbp pan-genome is highly concordant with the phylogeny based on the SNPs found in the conserved core of the same strains (**Figure 5**). In both trees the serovars cluster together and in the same relation to each other, for example serovars Typhi and Paratyphi strains form a discrete monophyletic clade. However, the branch lengths in the pan-genome tree are larger than those in the conserved SNP tree, due to the larger variation among the presence/absence of the pan-genome than to sequence variation among shared core regions.

### Identification of a Minimum Set of Species-Specific Genomic Markers

Within the 404 species-specific markers, none were specific for any of the subspecies. That is, a marker was always present in genomes from at least two subspecies.

We next determined that the presence of a minimum set of two genomic regions was required to unambiguously identify genomes of *S. enterica*, within the 4893 genomes of the current study. A combination of two genomic regions were all that was required, and two such markers that were also present in the most *S. enterica* genomes were found at the following locations within the Typhimurium reference genome LT2: (1336001.. 1337000) and (2467001.. 2468000) (Supplementary File 3). All members of *S. enterica* examined contained at least one of these markers, but many other combinations within the 404 species-specific markers are also possible.

### Putative Functional Identification of the *S. enterica* Species-Specific Regions

The putative function of the 404 quality-filtered *S. enterica* species-specific regions were determined from the GenBank nr database. The annotation of each of the 404 regions is available as Supplementary File 1. **Table 4** summarizes the frequency of functional annotation categories, after annotating each region with the single best match. As can be seen, hypothetical proteins accounted for the majority (64) of the 404 annotations, with secreted effector and membrane proteins being the next most frequent category among the species-specific regions. Other membrane, transport, and secretion proteins were observed. The species-specific regions also included proteins involved in core metabolic functions, protein and DNA synthesis, and response to stress.

**Table 4:** The putative function of the *S. enterica* species-specific regions for functions that were identified more than once, utilizing the best hit for each region.

Putative protein function	Frequency
Hypothetical	64
Secreted effector	10
Membrane	7
Secretion system apparatus	5
Uncharacterized	5
Fimbrial	5
Pathogenicity island 2 effector	4
Fimbrial assembly	4
Outer membrane usher	4
mfs transporter	3
Oxidoreductase	3
Histidine kinase	3
Putative inner membrane	3
Putative cytoplasmic	3
lysr family transcriptional regulator	3
Transcriptional regulator	2
Permease	2
Outer membrane	2
Type III secretion	2
Phosphoglycerate transport	2
arac family transcriptional regulator	2
Conserved hypothetical	2
Methyl-accepting chemotaxis	2
Hybrid sensor histidine kinase/response regulator	2
Glycosyl transferase, partial	2
Phenylacetaldehyde dehydrogenase	2
Pathogenicity island 1 effector	2
*n*-Acetylneuraminic acid mutarotase, partial	2
Type III secretion system	2
Transcriptional regulator, partial	2
Cytoplasmic	2
Fimbrial chaperone	2
Putative sialic acid transporter	2


*The complete list of all putative functions is available as Supplementary File 3.*

### Identification of Subspecies-Specific Markers from the Pan-genome

Having identified species-specific markers, we employed the same techniques, utilizing the presence/absence of all pan-genome markers, just as was carried out in identifying the 404 species-specific ones, to identify subspecies-specific markers. The number of markers that were completely unique to a subspecies is given in **Table 5**. Subspecies *arizonae* contained the most unique markers, at 207, and *enterica* contained the least, at 9.

**Table 5:** The number of subspecies-specific pan-genome markers that were universally present or absent among members of the subspecies, and not absent or present among genomes from any other subspecies.

Subspecies	No. markers
*arizonae*	207
*diarizonae*	93
*enterica*	9
*houtenae*	134
*indica*	192
*salamae*	135


### Identification of Universal Serovar Markers within *Subspecies enterica* from the Pan-genome

Subspecies *enterica* genomes were the vast majority of those available, so we attempted to identify serovar-specific markers for the top 10 serovars, in the same manner that we identified subspecies-specific markers. We found that there were no genomic markers that uniquely defined any of the serovars based on their presence or absence; however, there were a number of genomic regions that were universally present or absent among serovars, as well as statistically over- or under- represented with respect to all other serovar genomes from this study; they are shown in **Table 6**.

**Table 6:** The number of pan-genome regions that were universally present and absent, as well as statistically over- or under-represented in comparison to all other genomes, within the 10 most abundant serovars within the 4870 subspecies *enterica* genomes of this study.

Serovar	No. universally present	No. universally absent
Typhi	288	2720
Typhimurium	41	698
Enteritidis	18	440
Heidelberg	121	840
Paratyphi	65	202
Kentucky	177	331
Agona	161	638
Weltevreden	426	608
Bareilly	87	436
Newport	226	360


**FIGURE 7:**
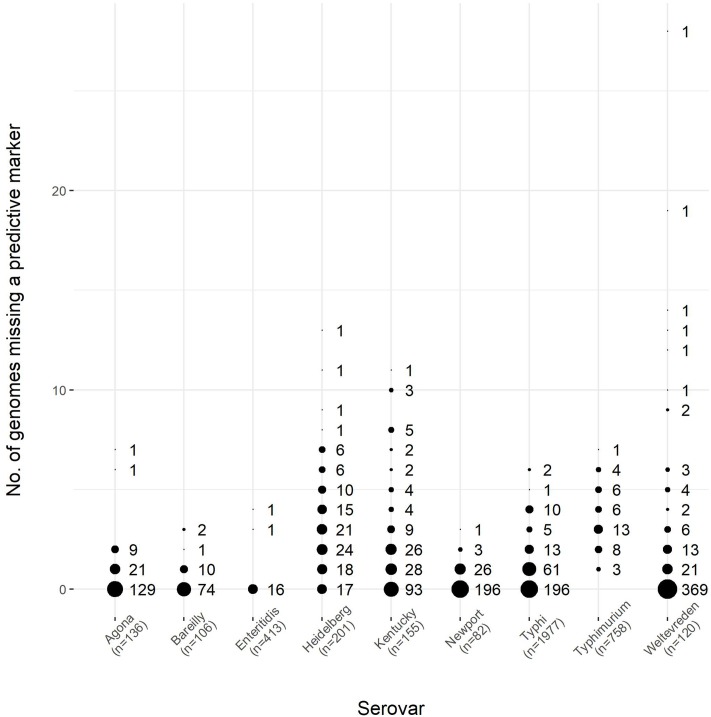
The number of predictive markers from the GenBank dataset found within the EnteroBase dataset for nine serovars of *S. enterica*, which encompassed a test set of 3948 genomes. The number of genomes for each serovar was the same between the GenBank and EnteroBase datasets, as shown in **Table 6**. The size of the circles is proportional to the number of predictive markers from the GenBank dataset found in the EnteroBase dataset. The number of genomes for each serovar is given in the horizontal axis label. Using serovar Agona as an example, there were 136 genomes in both the GenBank and EnteroBase datasets, and 129 of the 161 predictive markers from the GenBank dataset were found in all of the genomes from the EnteroBase dataset, whereas 21 of the GenBank predictive markers were found in all but one (135) of the EnteroBase genomes examined.

To further assess the validity of these markers, a dataset comprised of 3948 genomes from EnteroBase^2^, was selected to have an identical number of strains belonging to each of nine serovars in our GenBank dataset. The EnteroBase dataset was used to test the predictive markers we identified from the GenBank dataset in the first part of the study. The results of this comparison are shown in **Figure 7**. As can be seen, the markers were well-conserved among the EnteroBase dataset, with eight of the nine serovars having a subset of the predictive markers present among all of the test genomes; serovar Typhimurium had a marker subset that was present in all but one of the test genomes.

## Discussion

### *S. enterica* Pan-genome

Previous examinations of the *S. enterica* pan-genome were based on relatively small datasets of 45 and 73 genomes (Jacobsen et al., 2011; Leekitcharoenphon et al., 2012). While others have analyzed 1000s of *S. enterica* genomes, the analyses were not conducted to examine the population structure. For example, in demonstrating the software program Roary, 1000 *S.* Typhi genomes were used to test the program (Page et al., 2015). Likewise, the GenomeTrackR project utilized 32 *S. enterica* genomes to identify a *S. enterica* core, which was subsequently used as the basis for genetic distance estimates for nearly 20,000 genomes (Pettengill et al., 2016).

Previous estimates placed the core-genome size of *S. enterica* at ∼2800 gene families, and the pan-genome at ∼10,000 gene families (Jacobsen et al., 2011). The current study identified a strict core of 1.5 Mbp, and a conserved core of 3.2 Mbp shared among 96% of the genomes, which given an average gene size of 1000 bp is ∼1500 and ∼3200 genes respectively, with a much larger pan-genome of ∼25,300 genes. Previous analyses found *S. enterica* to have a closed pan-genome (Jacobsen et al., 2011), and thus the rate of discovery for new genomic regions would decrease for each new genome of the species sequenced (Tettelin et al., 2005).

In line with *S. enterica* having a closed pan-genome, when we compared it to *E. coli*, a related bacterial species with an open pan-genome (Tettelin et al., 2005), we found that the *E. coli* pan-genome was larger (37.4 Mbp), despite the fact that the *E. coli* study used less than half the number of strains in the current *Salmonella enterica* study. Additionally, more of the pan-genome of *S. enterica* was distributed among more genomes than in *E. coli* (Whiteside et al., 2016). Specifically, in *S. enterica* 70% of the pan-genome was found to belong to 100 or fewer of the genomes examined, while in *E. coli* 80% of the pan-genome was found in 100 or fewer genomes.

It should be noted that erroneously labeled, and poor quality assemblies, can greatly affect the size, analyses, and composition of the pan-genome. Software tools to evaluate assembly quality have been created to help researchers identify bad data. These include QUAST (Gurevich et al., 2013), which summarizes the assembly statistics including average contig size and number of contigs; as well as CGAL (Rahman and Pachter, 2013), which uses a likelihood approach to infer assembly quality rather than summary statistics. As demonstrated in the current study, having a known set of species-specific genome regions can facilitate rapid quality assessment and filtering of genome assemblies. Others have proposed whole-genome MLST for this purpose as well (Babenko et al., 2016; Yoshida et al., 2016b), but the benefit of a pan-genome analysis is that it is schema free, requiring no agreed upon reference set or central repository of alleles.

### *S. enterica* Species-Specific Regions

Previous studies have identified gene targets that are useful in the identification of *Salmonella*. These include the *fimA* gene (Cohen et al., 1996), *hilA* (Guo et al., 2000), *invA* (Malorny et al., 2003), *ttr* (Malorny et al., 2004), and *ssaN* (Chen et al., 2010). Other markers, and combinations thereof have been developed for use in RT-PCR (Postollec et al., 2011), and other detection platforms such as loop-mediated isothermal amplification (Kokkinos et al., 2014). Additionally, the identification of serovar based on allelic variation in somatic and flagellar genes has previously been conducted, with at least four laboratory methods currently available [the *Salmonella* genoserotyping assay (Yoshida et al., 2014), and the commerical assays: *Salmonella* Serogenotyping Assay, Check&Trace *Salmonella*, and xMAP *Salmonella* serotyping assay], capable of identifying over 100 of the most common *Salmonella enterica* serovars in some cases (Yoshida et al., 2016a). The recently released software, the *Salmonella in silico* typing resource (SISTR), is capable of providing *Salmonella* serovar prediction from WGSs for 90% (2,190) of all serovars (Yoshida et al., 2016b).

Despite the utility of the previously mentioned methods, previous marker-discover studies have used at most 100s of *Salmonella* strains, while the current study examines nearly 5000. Further, the current study analyzes the entire pan-genome for predictive markers, and identified over 400 that were specific to the species, as well as others being predictive for both subspecies and serovar.

The host intestinal environment consists of a multitude of bacterial species competing for scarce nutritional sources such as carbohydrates, direct antagonistic competition with other bacterial cells, and competition for access to the host intestine, where stable attachment and colonization of the local environment are possible (Sana et al., 2016). The normal intestinal microflora offer protection to the host against enteric pathogens such as *S. enterica*, but disruption of the intestinal environment by virulence factors and effector proteins secreted by the pathogen itself, or external factors including antibiotics, have been shown to alter the composition of the microbiota, and allow pathogens such as *S. enterica* to proliferate (Ng et al., 2013).

Nutritional competition exists for free metabolic compounds, such as carbohydrates that are readily available, as well as others that are sequestered in forms such as the intestinal mucus, which is composed of sialic sugar acids (McDonald et al., 2016). In the gut, these sugar acids exists as a conjugate in the alpha form, which to be useful for bacteria such as *Salmonella*, need to be converted to the beta form by a mutarotase enzyme (Severi et al., 2008). In this study, we identified n-acetylneuraminic acid mutarotase genes as species-specific genomic regions, along with sialic acid transporter genes. It is possible the presence of these systems allow *S. enterica* to more efficiently compete with the host microbiota by efficiently utilizing scarce metabolic sources.

It was also previously found that sialic acid on the surface of host colon cells increased colonization by *S.* Typhi, and disialylation of these cells reduced the adherence of the *Salmonella* strains by 41% (Sakarya et al., 2010). This was also demonstrated in *S.* Typhimurium, where following antibiotic treatment, the presence of free sialic acid increased, and the ability to utilize it was correlated with higher levels of bacterial colonization of the host gut (Ng et al., 2013).

Enzymes that utilize sialic acids have previously been shown to be present in 452 bacterial species, including other pathogens such as *Vibrio cholerae*, but the genomic regions found in the current study were sufficiently unique at the nucleotide level to be determinative for *S. enterica* (McDonald et al., 2016).

In addition to species-specific regions used to gain a metabolic advantage, a number of secretion system and effector proteins were identified as diagnostic of *S. enterica*. These included components of the Type VI secretion system (T6SS), which is a contact-dependent, syringe-like secretion system that allows *S. enterica* to directly kill other competing bacteria that it comes into physical contact with (Brunet et al., 2015), and is encoded on the *Salmonella* Pathogenicity Island 6 (Sana et al., 2016). It has been demonstrated that silencing the T6SS via H-NS repression (histone-like nucleoid structuring), reduces inter-bacterial killing of *S. enterica* (Brunet et al., 2015). It was also previously shown that commensal bacteria are killed by *S. enterica* in a T6SS-dependent manner, that the T6SS was required for *Salmonella* to establish infection in the host gut, and that increased concentrations of bile salts resulted in a concomitant increase in T6SS anti-bacterial activity (Sana et al., 2016). The T6SS itself has been shown to have been independently acquired from four separate lineages within five of the six *S. enterica* subspecies (Desai et al., 2013).

Like the T6SS, the type III secretion system (T3SS) found within *S. enterica* is a syringe like apparatus that injects effector proteins into host cells (Kubori et al., 2000). There are two T3SS found within *S. enterica*: the first is encoded on the *Salmonella* Pathogenicity Island 1 (SPI1) and is required for invasion of host cells; the second is encoded on *Salmonella* Pathogenicity Island 2 (SPI2), and is required for survival and proliferation within the host macrophage cells (Hensel et al., 1998; Bijlsma and Groisman, 2005). The innate host immune system utilizes the inflammatory response to help reduce the proliferation of bacterial pathogens (Sun et al., 2016). *S. enterica* has developed a means of regulating host inflammation via the SPI1 T3SS, whereby secreted effector proteins target the NF-κB signaling pathway, reduce inflammation and host tissue damage, and allow increased *S. enterica* propagation within the host. *S. enterica* also relies on free long-chain fatty acids within the host to regulate T3SS expression, and provide a cue to the bacteria to up-regulate genes necessary for host intestinal colonization (Golubeva et al., 2016).

The current study identified many secretion system and effector proteins as being species-specific, as well as proteins for attachment to the host, such as fimbriae. These proteins allow *S. enterica* to compete within the intestinal environment, and take up residence within the host, where it can proliferate.

Effector proteins and other virulence factors aid in the colonization of the host, and are frequently horizontally acquired and are present on mobile elements such as integrated bacteriophages (Moreno Switt et al., 2013). Previous work identified clusters of phages that carried virulence factors such as adhesins and antimicrobial resistance determinants within *S. enterica* (Moreno Switt et al., 2013).

Additionally, many of the genes associated with bacteriophage in *S. enterica* have been found to be of the putative and hypothetical class (Penadés et al., 2015). The current study identified a large accessory gene pool that contained many hypothetical and putative genes, which were also the most abundant category of species-specific genomic regions. The proteins of putative and unknown function may aid in colonizing warm-blooded animals, or specific animal or environmental niches. Previous studies identified genotype/phenotype correlations of *S.* Typhimurium that had particular gene complements associated with specific food sources (Hayden et al., 2016). The same study also postulated that specific phage repertoires may give phylogenetically distant strains a similar accessory gene content, and therefore similar niche specificity. Previously, 285 gene families were identified as being recruited into *S. enterica*, where most of these genes had unknown function, but were postulated to be important for its survival and infection of its host (Desai et al., 2013). It is therefore not surprising to find that the most abundant species-specific category of genomic regions are those of unknown or putative function; they likely represent genes enhancing the ability of *S. enterica* to propagate within warm-blooded animals, but they have not yet been fully characterized. The other genomic regions diagnostic of *S. enterica* include means for disseminating these fitness genes within the population, competing for resources in the host, and attaching and proliferating. The *S. enterica* species-specific regions likely give a good overview of the factors responsible for making it such an effective pathogen and intestinal inhabitant.

### Specific Regions for Subspecies and Serovar

The current study recapitulates the phylogenetic relationship of the six *S. enterica* subspecies that has been previously described by others (Desai et al., 2013). However, the number of species-specific regions found within each subspecies does not follow the same pattern. For example, *diarizonae* is more distantly related to *enterica* than subspecies *indica*, but contains more species-specific regions, and the branch lengths on the tree are shorter. This indicates that although the *diarizonae* strains diverged longer ago than the houtenae strains, they have accumulated less genomic change. Both subspecies *diarizonae* and *houtenae* strains are associated with reptile-acquired salmonellosis (Schroter et al., 2004; Horvath et al., 2016), but the differences in genomic change may reflect the specific reptile niches that each inhabit.

Genomic regions specific to each subspecies were identified, the presence of which were unambiguously indicative of each subspecies. The most abundant subspecies in the current analyses, *enterica*, had the fewest specific markers present (9), while the most distantly related subspecies *arizonae*, had the most specific markers (207). These results indicate that just as core genome size decreases with the number of genomes examined, so too do the number of markers “core” to each subspecies. As more genomes in subspecies *arizonae* and closely related subspecies are examined, we would expect fewer genomic regions to remain specific for the subspecies. This has important implications for designing a set of markers indicative for subspecies, indicating that a group of redundant markers should be used, and that a sampling of the diversity within a subspecies is first required to identify genomic regions that are truly core.

This was also observed within serovar for subspecies *enterica* strains. The original study examining the pan-genome of *S. enterica* used a set of 45 genomes and was able to identify unique gene families for each serovar examined, with Enteritidis having the fewest (29), and Typhi having the most (349) (Jacobsen et al., 2011). The results of the current study showed no unique genomic regions for any of the serovars with a sample set of 4893 quality filtered genomes. Although genomic regions universally present for each serovar were observed, and followed the same pattern with Enteritidis having the fewest (18), and Typhi having the most (288), these regions were also observed among genomes of other serovars, even though they were statistically over-represented for the serovar in question. The presence of these predictive markers in nearly all of the genomes within the EnteroBase test dataset indicates that the markers are robust, indicative of serovar, and could be combined to determine the likelihood of a genome being of a particular serovar.

When examining the average number of the 404 species-specific regions found among the *enterica* serovars, it was interesting to observe that Enteritidis, which had the fewest number of universal genomic regions, had the highest average number of species-specific regions; likewise Typhi, which had the most universally shared genomic regions, had one of the lowest averages of species-specific regions present. These results indicate that Enteritidis is the serovar that is closest to being the “core” example of a *S. enterica* genome, while Typhi is the serovar that is the most divergent. *S.* Enteritidis is the most common cause of enteric *Salmonella* infection, causing upward of one quarter of all infections, and is prevalent in chickens as well as their eggs (Chai et al., 2012). Conversely, *S.* Typhi is a human adapted serovar, responsible for Typhoid fever, and observed to have undergone genome degradation, rearrangement, and acquisition through horizontal gene-transfer, as it has evolved within its human host (Sabbagh et al., 2010; Klemm et al., 2016). It thus appears that genomic change enabling adaptation to a host creates a genomic pool that distinguishes a group from others of the same species. At the same time, genetically similar serovars that maintain a broad host range do not undergo as much selection for genomic change are much harder to distinguish as separate groups, but much easier to identify as members of the subspecies.

### Core and Pan-genome Comparison

Most phylogentic studies focus on variation within homologs in the core genome to infer evolutionary relationships (Treangen et al., 2014), as paralogs and horizontally transferred elements confound the evolutionary signal found in genes obtained through vertical descent over time (Gabaldón and Koonin, 2013). While this approach is undoubtedly useful for long-term evolutionary analyses, when attempting to identify phenotypic linkages between phylogenetic clades, the accessory genome needs to be taken into account, as non-ubiquitous genomic regions allow different groups within the species to occupy and thrive in specific niches (Polz et al., 2013). Additionally, it has recently been shown that regulatory switching to non-homologous regulatory regions acquired via horizontal gene transfer happens in many bacteria (Oren et al., 2014). It was further shown that regulatory regions can move without the genes they regulate moving, and that at least 16% of the differences in expression observed within an *E. coli* population were explained by this regulatory switching.

It is therefore prudent to examine both the accessory genome, and not just genes, but non-coding DNA as well, as both have been shown to influence gene expression, and niche specificity. Recent studies have shown that the concordance between a phylogeny based on core genome SNPs and the presence/absence of pan-genome regions is high. For example, in a study examining *E. coli* lineage ST131, the core and accessory genomes showed high concordance, and the combined analyses of both allowed the analyses of the evolution of the *E. coli* lineage at a resolution not possible if only a restricted portion of the genome had been considered (McNally et al., 2016). The current study shows the same concordant relationship within *S. enterica* between the core and accessory genome, indicating that the accessory genome is not just randomly acquired genomic material, but that selection within specific niches establishes a complement of genes and regulatory elements that enable the survival of the *S. enterica* strains present. It also suggests that to understand why particular clades are more virulent, or possess a particular phenotype, a pan-genomic approach should be used in comparative analyses.

## Conclusion

We examined a quality filtered set of 4893 genomes, the largest pan-genomic study of the *S. enterica* species to date. We identified a pan-genome of 25.3 Mbp, a strict core of 1.5 Mbp present in all genomes, and a conserved core of 3.2 Mbp found in at least 96% of the genomes in this study. In addition we identified 404 species-specific regions, within which a minimum set of two was required to unambiguously identify a genome as being part of the species *S. enterica*. These species-specific regions were found to have functions related to the propagation in and colonization of the host, including the utilization of sialic acid in intestinal mucus, secretion systems for attachment to the host, and the killing of other host microbiota. Within subspecies *enterica*, the species-specific regions were found most frequently in serovar Enteritidis. Each of the six subspecies was found to have genomic regions specific to it; however, the number of subspecies-specific regions appeared to be correlated with the level of sampling of the diversity within the subspecies. No serovar had pan-genome regions that were present in all of its representative genomes and absent in all other serovar genomes; however, each serovar did have genomic regions that were universally present among all constituent members, and statistically predictive of the serovar. *S.* Typhi, which is host-adapted to humans, was found to have the most universal markers predictive of its serovar. The phylogeny based on SNPs within the conserved core genome was found to be highly concordant to that produced by a phylogeny using the presence/absence of the entire pan-genome, and both agreed with phylogenies previously reported for *S. enterica*. Together, the core and accessory genome offered a more complete picture of the diversity within the genomes than either alone. The genomic regions identified in this study that are predictive of the species *S. enterica*, its six subspecies, and the serovar groups within subspecies *enterica*, could be developed into simple and rapid diagnostic tests, with uses ranging from food safety to public health. Additionally, the tools and methods described in this study could be generally applicable as a pan-genomics framework for future population studies, or those looking for genotype/phenotype linkages.

## Author Contributions

CL: designed the experiments, analyzed the data, and wrote the manuscript. MW: designed the experiments, and wrote the manuscript. VG: designed the experiments, and wrote the manuscript.

## Conflict of Interest Statement

The authors declare that the research was conducted in the absence of any commercial or financial relationships that could be construed as a potential conflict of interest.

## Footnotes

1 https://github.com/chadlaing/feht

2 https://enterobase.warwick.ac.uk/species/index/senterica
